# Double Deception: Ant-Mimicking Spiders Elude Both Visually- and Chemically-Oriented Predators

**DOI:** 10.1371/journal.pone.0079660

**Published:** 2013-11-13

**Authors:** Divya Uma, Caitlin Durkee, Gudrun Herzner, Martha Weiss

**Affiliations:** 1 Department of Biology, Georgetown University, Washington, D. C., United States of America; 2 School of Biology, Indian Institute of Science Education and Research, Trivandrum, India; 3 Department of Zoology, University of Regensburg, Regensburg, Germany; University of Tours, France

## Abstract

Biological mimicry is often multimodal, in that a mimic reinforces its resemblance to another organism via different kinds of signals that can be perceived by a specific target audience. In this paper we describe a novel scenario, in which a mimic deceives at least two distinct audiences, each of which relies primarily on a different sensory modality for decision-making. We have previously shown that *Peckhamia picata*, a myrmecomorphic spider that morphologically and behaviorally resembles the ant *Camponotus nearcticus*, experiences reduced predation by visually-oriented jumping spiders. Here we report that *Peckhamia* also faces reduced aggression from spider-hunting sphecid wasps as well as from its model ant, both of which use chemical cues to identify prey. We also report that *Peckhamia* does not chemically resemble its model ants, and that its total cuticular hydrocarbons are significantly lower than those of the ants and non-mimic spiders. Although further studies are needed to clarify the basis of *Peckhamia's* chemically-mediated protection, to our knowledge, such ‘double deception,’ in which a single organism sends misleading visual cues to one set of predators while chemically misleading another set, has not been reported; however, it is likely to be common among what have until now been considered purely visual mimics.

## Introduction

Biological mimics can employ a range of signals to achieve deceit: male *Andrena* bees are duped into attempting to mate with *Ophrys* orchids by the flowers' remarkably accurate chemical, visual and textural resemblance to a female bee, and predatory lycaenid caterpillars gain access to the nests of *Myrmica* ants by mimicking the ants' characteristic chemical and acoustic signals [1]. In these examples, a mimic reinforces its resemblance to another organism via multiple signals that can be perceived by a specific target audience. Here we describe a novel scenario, in which a mimic uses different kinds of sensory information to simultaneously deceive at least two distinct audiences, each of which relies primarily on a different sensory modality for decision-making.

Ants are common models for numerous mimetic arthropods, as these aggressive, noxious, social insects are typically avoided by generalist predators [2]–[3]. Ant-like appearance (myrmecomorphy) is found in insects and spiders belonging to over 200 genera in 54 families, in groups as diverse as beetles, mantids, true bugs, crickets, and spiders [3]. Because myrmecomorphy is particularly common amongst spiders, occurring in 13 taxonomically widespread families [4], most experimental work on the phenomenon has focused on this group. Spiders' morphological and behavioral resemblance to ants can range from merely suggestive to astonishingly accurate [5]–[7]. Mimetic features seen in spiders often include a constricted mid-body that resembles an ant's narrow ‘waist,’ darkly pigmented regions on the head that suggest compound eyes, and waving of front legs in an ‘antennal illusion’ [3]–[4].

Myrmecomorphs are commonly presumed to be Batesian mimics [3]–[4], [8]–[11]; that is, a palatable arthropod's resemblance to an unpalatable ant confers protection against predation by visually-oriented predators. Recent studies have shown that ant-mimicking spiders do indeed deceive mantids and jumping spiders, both of which base their foraging decisions on visual cues [6], [12]–[15].

Although morphological and behavioral resemblance to ants confers protection against visual hunters, myrmecomorphic spiders also encounter chemically-oriented predators, which are unlikely to be dissuaded by the spiders' ant-like appearance. Are they protected against these potential predators, and if so, how?

Many wasps rely on chemical cues to identify their prey [16]–[19]. In particular, mud-dauber wasps are major predators of spiders, as females provision each of their many larval cells with approximately a dozen paralyzed spiders [20]–[21]. The wasps predominantly capture spiders that build two-dimensional orb webs, but are also known to take non-web-building spiders, including jumping spiders. Interestingly, myrmecomorphic jumping spiders are rarely found in wasp nests, and it has been suggested that these spiders escape wasp predation as a result of their visual resemblance to ants [10], [22]. However, we have demonstrated that while the spider-hunting wasp *Sceliphron caementarium* uses visual cues to locate potential prey items, foragers rely entirely on contact chemosensory cues to recognize suitable spiders [19]. Indeed, foraging wasps will touch and sting a paper ball coated with cuticular extracts of a prey spider species, but will touch and subsequently ignore visually identical balls coated with extracts from non-prey spiders [19]. Thus it is possible that myrmecomorphic spiders are not recognized by wasps as prey because the mimics lack prey-specific chemical profiles that are present on other, non-mimetic spiders.

Spider-hunting wasps are not the only chemically-oriented predators of myrmecomorphic spiders. The mimics are also vulnerable to attack from their own model ants, as both model and mimic are generally found within the same habitat [4], [23]. Ants have been described as ‘walking chemical factories’ [2], since many species rely primarily on information contained in cuticular hydrocarbons for various task decisions [24], including recognition and discrimination of nestmates from non-nestmates, foraging, and nest construction [24]–[26]. Thus it is likely that the cuticular chemistry of spiders living in close association with ants will also be under selection by the ants [23], [27].

**Figure 1:**
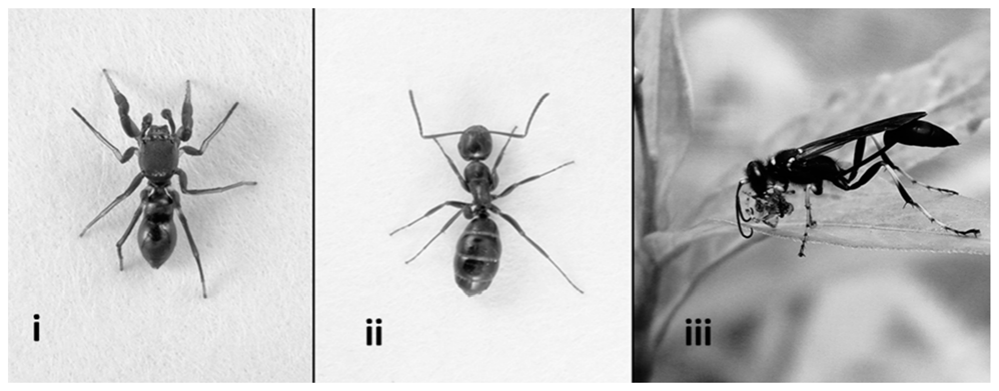
Ant-mimicking spider and two potential predators: i) *Peckhamia picata*, ant-mimicking jumping spider; ii) *Camponotus nearcticus*, putative ant model and a predator; iii) *Sceliphron caementarium*, spider-hunting predatory wasp. (Figures not to scale. Ant and ant-mimic photos by J. Coddington, wasp photo by D.Uma).

In previous work we have shown that *Peckhamia picata* (Salticidae), a myrmecomorphic jumping spider (Fig. 1A), is significantly less likely to be captured by other visually-oriented predatory jumping spiders than are non-mimetic spiders [6]. Here, we use behavioral assays to examine the responses of chemically-oriented wasps and ants to *Peckhamia picata* (henceforth *Peckhamia*). Specifically, we asked whether *Peckhamia* elicits aggressive behavior either from spider-hunting mud-dauber wasps, or from its own model ants. We also characterized the cuticular chemistry of *Peckhamia*, its ant model, and a non-mimetic jumping spider, in order to evaluate the potential for chemical deception.

## Materials and Methods

### Ethics statement

Specimens were collected on land belonging to Georgetown University and along the C & O Canal, with a permit from the National Park Service. Our field studies did not involve endangered or protected species.

### Study organisms

#### Spiders

We collected our focal ant-mimicking spider, *Peckhamia picata*, as well as non-mimetic salticids (*Paraphidippus aurantius, Phidippus putnami*) from weedy habitat near the Potomac River, Washington DC, between May and July in 2008–2011. Spiders were sub-adults of both sexes. Animals were located by visual search, and most were found on or around a metal fence railing. Individual spiders were housed separately in a small covered opaque plastic cup (32 ml volume) containing a moist cotton ball and a leaf for a substrate. Every other day, the spiders were fed two to three *Drosophila*.

#### Mud-dauber wasps

Female *Sceliphron caementarium* (Sphecidae, henceforth *Sceliphron*) wasps were collected in the Washington, D.C. area as they gathered mud for nest construction during the summers of 2008 and 2009. Individually marked wasps were housed 4 or 5 to an outdoor mesh cage (2 m^3^). Wasps were fed daily with honey-water, and the cages were provisioned with wooden shelters and wet mud for nest construction (See [19] for specifics of wasp maintenance).

#### Ants

We considered *Camponotus nearcticus* worker ants (henceforth *Camponotus*) the model for *Peckhamia*, as these ants were sympatric, abundant, and appeared most similar to the mimic in terms of size and color. We collected *Camponotus* worker ants from an ant trail on or near the same metal fence railing from which we collected *Peckhamia*, in summer 2010 and 2011. All ants collected from a single trail were considered ‘nestmate’ ants (relative to the focal ants, which were also taken from that group); ants of the same species collected on the campus of Georgetown University, approximately one mile from the Potomac River site, were considered ‘non-nestmate’ ants. Ants were collected the day before we used them in an experiment, and each was housed separately in a small covered opaque plastic cup (32 ml volume).

### Does *Peckhamia* elicit predatory behavior from spider-hunting mud-dauber wasps?

To determine whether *Peckhamia* elicited predatory behavior from spider-hunting mud-dauber wasps, we staged encounters between free-flying wasps and freshly killed mimetic and non-mimetic spiders. Once a wasp had built a mud cell inside the mesh cage, we temporarily enclosed the other wasps in a smaller mesh cage, and trained the focal wasp to fly down to a ‘testing arena’ [19]. There we presented her with paired freshly killed mimetic *(Peckhamia)* and non-mimetic *(Phidippus putnami)* spiders (frozen for 15 min @ −4C to control for movement) of comparable weight (average weight ±SD: mimic = 0.003±0.001 g, non-mimic = 0.002±0.002 g), positioned six cm apart on a filter paper disc (11 cm diameter). The paper disc itself was placed on a plastic mat (0.5 cm x 7 cm x 24 cm) located on the testing arena. Previous studies have shown that *Sceliphron caementarium* wasps respond similarly to live and freshly killed spiders, as the latter still have intact cuticular chemicals [19]. Each trial (n = 8), which consisted of an individual wasp encountering a pair of freshly-killed mimic and non-mimic spiders, commenced when a wasp walked or flew to the paper disc and ended when the wasp antennated both of the spiders and subsequently either stung or rejected them, or until 30 minutes had elapsed, whichever happened first. Each wasp was used only once in a single encounter with test mimic and non-mimic spiders, so there was no opportunity for learning to influence the results of subsequent tests. New spiders were used in each trial, their positions were switched between trials, and a fresh paper disc was used each time. Number of mimic and non-mimic spiders stung by wasps was analyzed using a Binomial Probability test. Additionally, in two instances, we observed foraging wasps' encounters with live ant-mimicking spiders.

### Does *Peckhamia* elicit aggressive behavior from its model ants?

To determine whether *Peckhamia* elicited aggressive behavior from its model ants, we staged sequential paired no-choice encounters between a single focal *Camponotus* ant and 4 different individuals — a mimetic jumping spider (*Peckhamia*), a non-mimetic jumping spider (*Paraphidippus aurantius*), a *Camponotus* nestmate ant, and a *Camponotus* non-nestmate ant. The test ants were included in order to provide additional comparative measures of aggression, as many ants, including *Camponotus*, typically show aggression towards non-nestmates, but not towards nestmates [26]. Spiders were matched for size (mean length of mimic  = 4.2±0.27 cm; non-mimic  = 4.3±0.44 cm), and the order of presentation of ants and spiders was randomized. The experiments were done blind with respect to the ants, as the collected ants were simply numbered, and not labeled as nestmate or non-nestmate. Trials (n = 25) were conducted in a 6 cm diameter plastic Petri dish, and each series of 4 interactions (treatments) was considered a single trial. After a focal ant had acclimated in the dish for 2 mins, we introduced one of the test subjects, and recorded the total number of contacts made and the total number of bites delivered by the focal ant towards the test subject for 5 mins. ‘Contact’ was defined as the focal ant antennating any part of the test subject. ‘Bite’ was defined as the focal ant coming in contact with a test subject with its mandibles open. Treatments were separated by a ten-minute window. Petri dishes were cleaned with ethanol between treatments to remove any residual chemicals and dragline silk [13]. A Panasonic 42× Optical Zoom video camera was used to record the encounters; two people independently scored number of contacts and bites in the videos. We used a Friedman's repeated measures ANOVA, followed by Bonferroni-Dunn's post-hoc tests (determined *a priori*), to compare the average number of contacts and bites by focal ants targeted towards test subjects. Specifically, we compared the responses of focal *Camponotus* ants to mimic *vs* non-mimic spiders, and to nestmate *vs* non-nestmate ants.

### Characterization of the cuticular chemistry of mimic, model ant, and non-mimetic spider

In order to explore possible mechanisms by which *Peckhamia* might escape aggression or predation by chemically-oriented enemies, we first characterized the cuticular chemistry of *Peckhamia*, *Camponotus*, and a non-mimetic jumping spider, and then compared their total amounts of surface hydrocarbons.

We obtained chemical profiles of model ants (n = 5), non-nestmate ants (n = 4), mimetic spiders (n = 5), and non-mimic spiders (*Paraphidippus aurantius*) (n = 5) by high-resolution gas chromatography - mass spectrometry (GC-MS, see below). Ants and spiders were individually surface-washed in 200 ul n-Hexane (Sigma Aldrich) for 10 min. One extract of each species was reduced in volume to approximately 2 µl and analyzed by GC-MS to determine the nature and quantity of internal standard necessary for quantification of the hydrocarbons. Based on these preliminary findings, heneicosane (C21) was added to all other extracts as an internal standard (0.05 µg (5 µl of a solution with the concentration 1 mg C21/100 ml hexane) to each vial) and the extracts were reduced in volume and analyzed by GC-MS. Thus, one less sample was used in the quantitative than the qualitative analysis.

GC-MS analysis was performed with an Agilent 6890N Series gas chromatograph (Agilent Technologies, Böblingen, Germany) coupled to an Agilent 5973 inert mass selective detector. The GC was fitted with an RH-5 ms+ fused silica capillary column (30 m×0.25 mm ID; film thickness = 0.25 µm, Capital Analytical, Leeds, England). The GC was programmed from 70 to 180°C at 30°C/min and then at 5°C/min to 310°C, with a 1-min initial isothermal and a 10-min final isothermal hold. A split/splitless injector (250°C) was used with the purge valve opened after 1 min. Helium was the carrier gas at a constant flow rate of 1 ml/min. Electron ionization mass spectra (EI-MS) were recorded at an ionization voltage of 70 eV, a source temperature of 230°C, and an interface temperature of 315°C. Data acquisition and storage were performed with the GC-MS software MSD ChemStation for Windows (Agilent Technologies, Palo Alto, CA, USA). Peak areas were obtained by manual integration using the GC-MS software.

Identification of n-alkanes was accomplished through comparison of their mass spectra with those of authentic reference compounds. Alkenes and alkadienes were identified by their typical mass spectra and linear retention indices. Methyl-branched hydrocarbons were identified by diagnostic ions resulting from their typical cleavage at the branching positions and by a fragment at M-15, when the molecular ion was not apparent. Additionally, their linear retention indices were calculated, and structural proposals were verified [28]–[29].

We performed multivariate statistical analyses to compare the cuticular profiles of model ants, non-nestmate ants, and mimics; our null hypothesis was that their chemical profiles did not differ. The total peak area of each individual extract was standardized to 100% and the relative areas of all peaks were calculated. We visualized differences in the chemical profiles between model ants, non-nestmate ants and mimics by non-metric multidimensional scaling (nmMDS), based on Bray-Curtis similarity measures [30]–[31] using the software PAST (version 2.10, [32]). In an MDS plot inter-point distances match the rank order of dissimilarities between samples in the underlying similarity matrix; deviations from a perfect match are expressed in terms of ‘stress,’ with stress values <0.15 indicating a meaningful representation of the data [33]. The significance of the differences between groups was assessed by one-way ANOSIM (ANalysis Of SIMilarity).

Because, in addition to the qualitative nature of cuticular chemicals, their total quantity may also provide recognition cues [34]–[36], we also compared the absolute amounts of surface hydrocarbons on ant (*Camponotus*), mimic spider (*Peckhamia*) and non-mimic spider (*Paraphidippus aurantius*). The absolute amount of each compound was determined by relating individual peak areas to the internal standard, and the total amount of hydrocarbons on each individual was calculated. The cuticular surface of each individual was estimated according to [36], using mathematical formulae for standard geometric shapes (text S1 in file S1), and the amount of cuticular hydrocarbon present per unit area was determined by dividing the total amount of CHCs by the cuticular surface of respective individuals. We used a Mann-Whitney U test to compare the size-corrected amounts of cuticular hydrocarbons. We chose to use *P. aurantius*, a non-mimic jumping spider that is found in the same habitat as *Peckhamia* but belongs to a closely related spider sub-family (Dendryphantinae), because all species belonging to the genus *Peckhamia* as well as its subfamily, *Synagelinae*, are ant mimics.

## Results

### *Peckhamia* does not elicit predatory behavior from spider-hunting mud-dauber wasps

**Figure 2:**
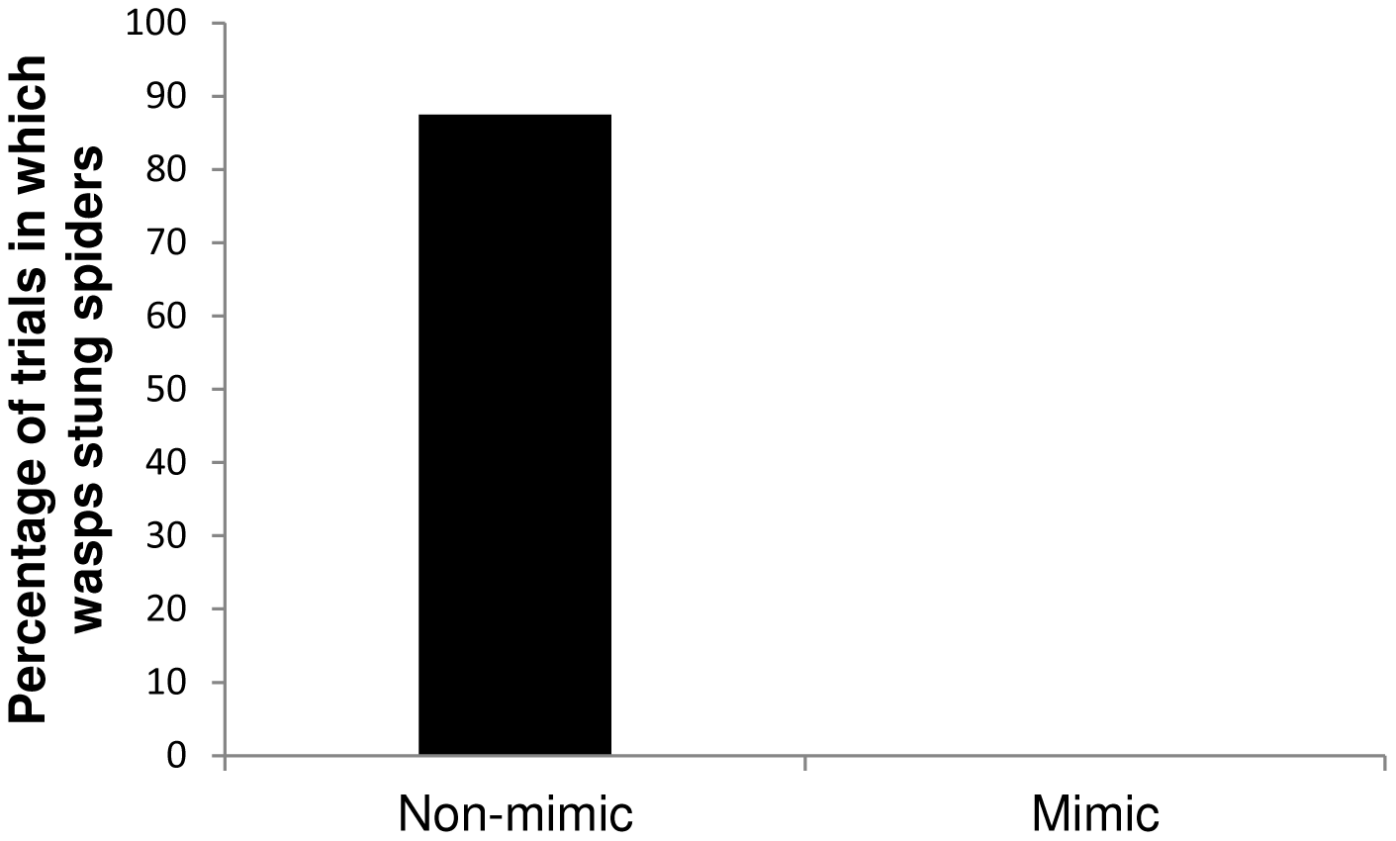
Ant-mimicking spider does not elicit predatory response from chemically-oriented spider-hunting wasps: *Sceliphron caementarium* wasps readily stung non-mimetic jumping spiders (*Phidippus putnami*) but never stung ant-mimicking spiders (*Peckhamia picata*) (binomial probability, P = 0.035).

When offered a simultaneous choice of freshly-killed, weight-matched mimic (*Peckhamia*) and non-mimic (*Phidippus putnami*) spiders, individual *Sceliphron* wasps antennated both spiders in eight pairs; they stung and captured seven of the eight non-mimics, but did not sting any of the mimics (binomial probability, P = 0.035, N = 7, Fig. 2).

### *Peckhamia* does not elicit aggressive behavior from its model ants

**Figure 3:**
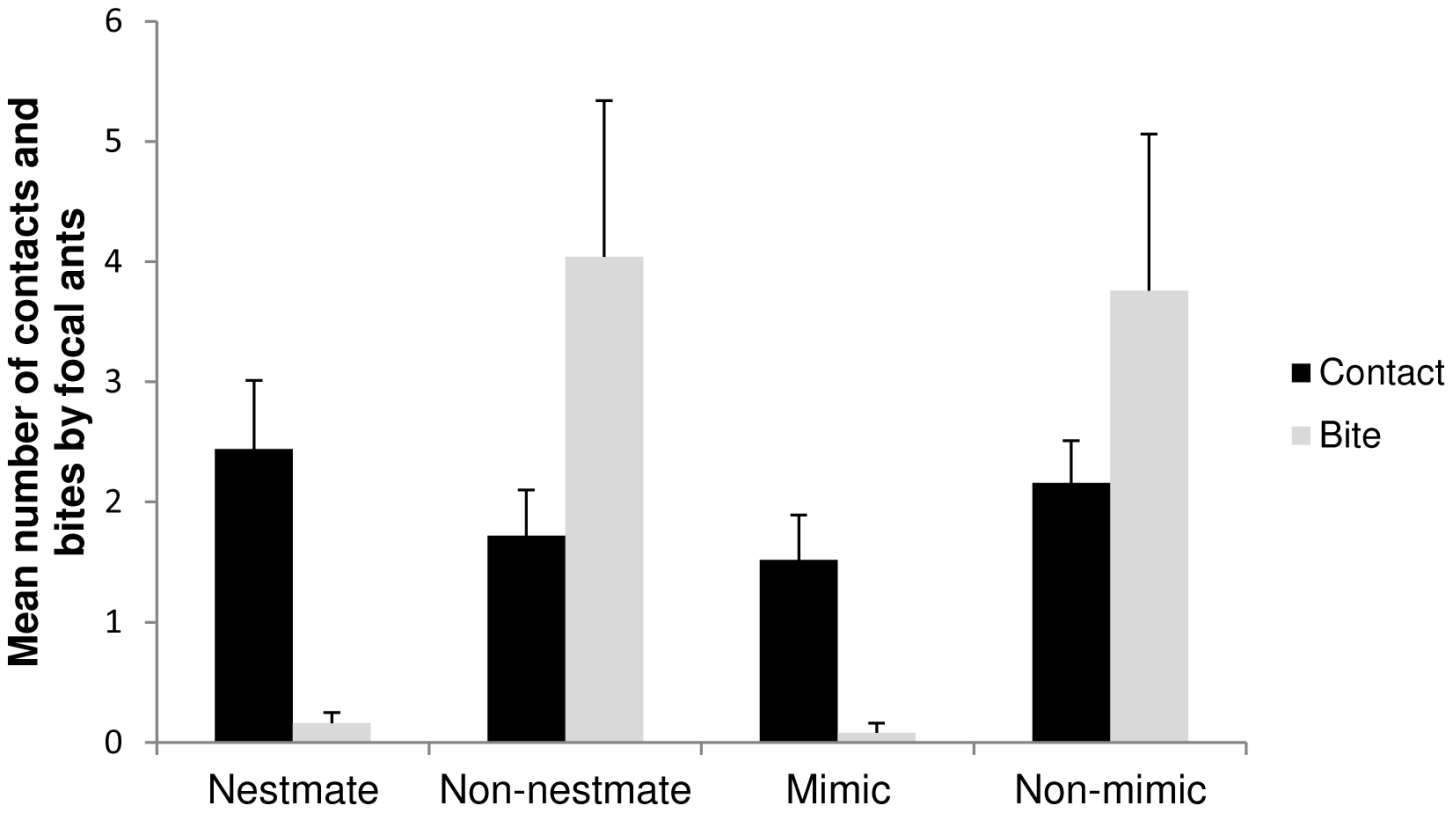
Ant-mimicking spider does not elicit aggressive response from ants: *Camponotus nearcticus* ants contacted all test subjects equally (Friedman non-parametric ANOVA, F = 1.71, P = 0.63 d.f. = 3), but displayed differences in aggressive response towards them (Friedman non-parametric ANOVA, F = 31.12, P = 0.0001, d.f. = 3). Ants also bit non-nestmate ants significantly more often than they bit their own nestmates (Bonferroni-Dunn test, P<0.01). In addition, ants bit non-mimic spiders significantly more often than they bit ant-mimic spiders (Bonferroni-Dunn test, P<0.05).

Focal *Camponotus* ants contacted nestmate ants, non-nestmate ants, and mimic and non-mimic spiders equally often, but displayed dramatic differences in their aggressive responses towards these test subjects (Fig. 3). Although the average number of contacts made by focal ants did not vary across the test subjects (Friedman non-parametric ANOVA, F = 1.71, P = 0.63 d.f. = 3) the ants' aggressive behavior towards the subjects varied significantly (Friedman non-parametric ANOVA F = 31.12, P = 0.0001, d.f. = 3). Specifically, the focal ants bit non-mimicking spiders significantly more often than they bit ant-mimicking spiders (Bonferroni-Dunn test, P<0.05). In addition, the focal ants also bit non-nestmate ants significantly more often than they bit their own nestmates (Bonferroni-Dunn test, P<0.01,Table S2 in file S1).

### *Peckhamia* is not a chemical mimic of ants, but has reduced amounts of cuticular hydrocarbons

The chromatograms of *Camponotus'* cuticular chemicals showed 34 peaks representing 41 compounds, with chain lengths between 25 to 31 carbon atoms (Table S3 in file S1). The chemical profile was dominated by monomethyl- and dimethylalkanes, with 11,13-dimethylheptacosane and 13,17-dimethylnonacosane being the most abundant compounds.

The samples of *Peckhamia* sub-adults revealed 48 peaks representing 71 compounds, ranging in chain length from 22 to 33 carbon atoms (Table S4 in file S1). The chemical profile showed mostly monomethylalkanes, with 2-methylhexacosane as the most abundant compound.

**Figure 4:**
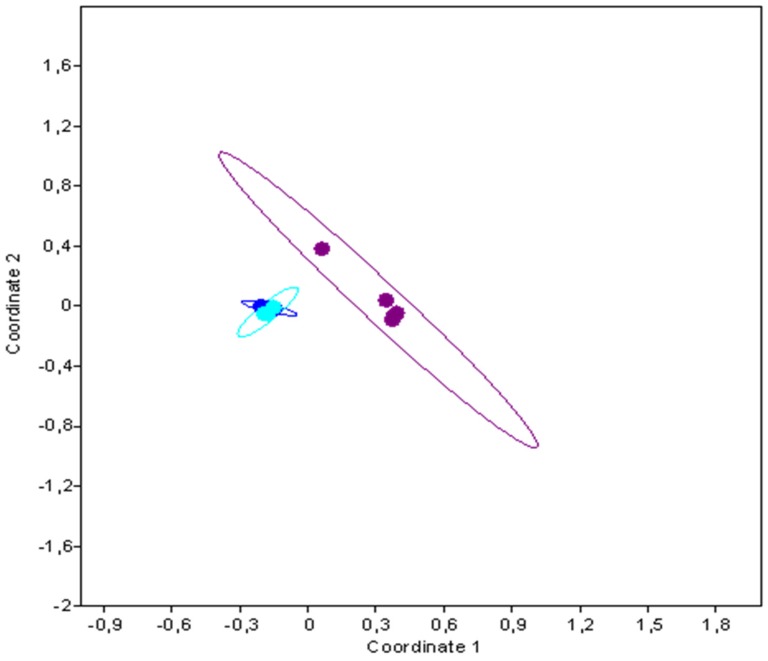
Ant-mimicking spider (*Peckhamia picata*) is not a chemical mimic of its model ant (*Camponotus nearcticus*). Two-dimensional non-metric multidimensional scaling plot of chemical profiles of mimic and ants (Stress value: 0.11). Purple dots represent ant mimic profile, dark blue dots represent model ant profile, and light blue dots represent non-nestmate ant profile.

The transformed peak areas were subjected to ordination analyses as described above. The profiles of the cuticular hydrocarbons of ants and mimics showed strong deviations, suggesting that *Peckhamia* is not a chemical mimic of *Camponotus* (Fig. 4, two-dimensional MDS plot; stress value: 0.11). As expected, however, the hydrocarbon profiles of nestmate and non-nestmate ants were quite similar to one another. The ANOSIM showed that the profiles of mimics, nestmate and non-nestmate ants were significantly different (R = 0.57, P = 0.001). Pairwise comparisons with sequential Bonferroni correction revealed significant differences between the mimic and both ant groups (mimic vs. nestmate ant, P = 0.006; mimic vs. non-nestmate ant, P = 0.015), and a non-significant difference between ant nestmates and non-nestmates (P = 0.07).

**Figure 5:**
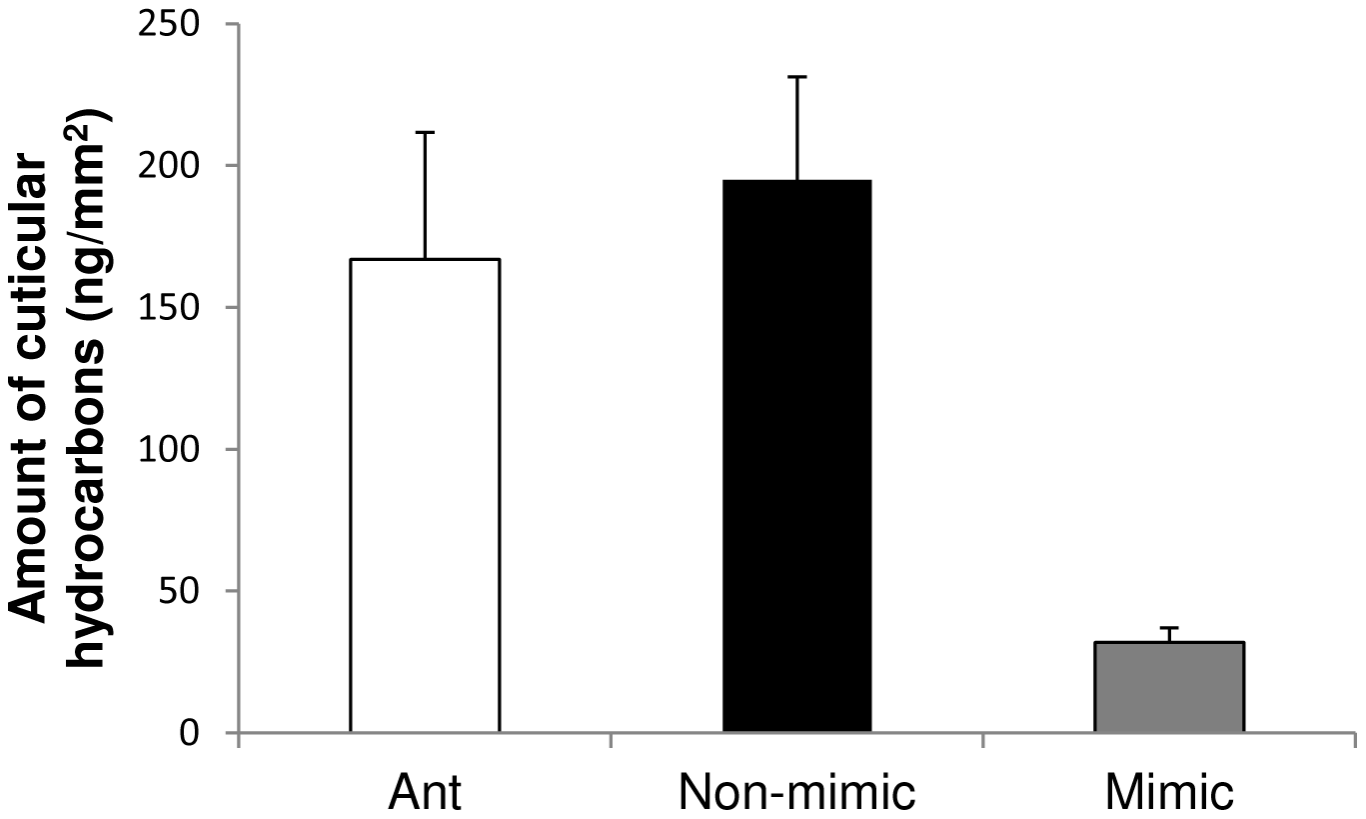
Ants (*Camponotus nearcticus*) and non-mimic spiders (*Paraphidippus aurantius*) have significantly higher levels of cuticular hydrocarbons than do mimics (*Peckhamia picata*): Ant vs. mimic, Mann-Whitney U = 20, Z = 2.4, P 0.015; Non-mimic vs. mimic: Mann-Whitney U = 16, Z = 2.3, P = 0.014).

Although *Peckhamia* is unlikely to be a chemical mimic of *Camponotus*, GC-MS analysis revealed that *Peckhamia*'s cuticular hydrocarbon levels are six-fold lower than those of the non-mimetic spider, *Paraphidippus aurantius* and the model ant *Camponotus* (mimic vs. non-mimic: Mann-Whitney U = 16, Z = 2.3, P = 0.014, mimic vs. ant: Mann-Whitney U = 20, Z = 2.4, P = 0.015, Fig. 5).

## Discussion

There is abundant evidence that ant-mimicking spiders in general gain protection from visually-oriented predators [12], [13]-[15], and we have shown specifically that *Peckhamia*, the subject of this study, experiences reduced predation from visually-oriented jumping spiders [6]. Our current results demonstrate that *Peckhamia* also avoids aggression from chemically-oriented predators, including mud-dauber wasps and ants. Thus, *Peckhamia* appears to engage in ‘double deception,’ as it eludes different audiences that rely upon distinct sensory modalities to recognize potential prey.

### Mimics do not elicit a predatory response from mud-dauber wasps

Spider-hunting sphecid and pompilid wasps typically use visual cues for long-range orientation, and chemical cues (olfactory or chemotactile) for near-field identification (sphecid wasps: [37], [16]–[17], [19]; pompilid wasps: [18], [38]. In earlier work, we found that *Sceliphron* wasps use visual cues, including movement and contrast, to identify an object as a potential prey item from a distance of 5–10 cm; at close range, however, they rely on chemotactile cues to determine suitability of the prey item [19]. Corroborating the importance of chemical information for prey recognition, *Sceliphron* wasps readily antennate and sting molts of non-mimetic spiders (species that they take as prey), and even paper balls coated with extracts of prey cuticular chemicals, but do not sting non-mimetic spiders from which the cuticular chemicals have been removed [19], [39].

When we offered wasp foragers a simultaneous choice between freshly-killed mimetic and non-mimetic jumping spiders, the wasps antennated both, but stung only the non-mimics. *Sceliphron* behaved similarly when we introduced two live *Peckhamia* onto a plant inside the field cage; two foraging wasps followed the moving mimics on the plant and antennated, but did not sting them. That the wasps rejected both live and freshly-killed mimics only after contacting them demonstrates that *Sceliphron* is not dissuaded by *Peckhamia's* ant-like morphology or behavior, but relies instead on chemical information for prey recognition.

### Mimics do not elicit aggressive responses from ants

Our behavioral results demonstrate that although *Camponotus* ants contacted both mimic and non-mimic spiders with the same frequency, the ants bit the mimics significantly less often than they did the non-mimics. Reduced ant aggression towards the mimics could result from a combination of chemical and/or behavioral traits in the mimics. It is possible that *Peckhamia's* low levels of cuticular hydrocarbons fell below the threshold perceptible to ants (chemical insignificance; see [40]–[41], although this hypothesis remains to be tested behaviorally. It is also possible that *Peckhamia's* behaviors help it to escape ant aggression. We observed that *Peckhamia* were more agile than non-mimics, and were quick to move away after being antennated by ants. Indeed, Nelson et al. [23] attribute the survival of myrmecomorphs in the presence of ants to the spiders' visual acuity, agility, and texture. Pekar & Jiros [27] suggest that the speed of movement of mimics is much greater than that of non-mimics following a disturbance.

### *Peckhamia's* cuticular chemistry

We have established that *Peckhamia* is *not* a chemical mimic of *Camponotus*. This result corroborates those of a recent study [27], which concluded that the cuticular hydrocarbon profiles of 5 species of ant-mimic spiders were not similar to the hydrocarbon profiles of their respective ant models. Thus, reduced frequency of bites by *Camponotus* is unlikely due to chemical mimicry by *Peckhamia*.

Furthermore, the quantity of cuticular hydrocarbons present on *Peckhamia* is one-sixth of that present on non-mimic spiders and one-fifth of that present on the ants themselves. Our preliminary analyses suggest that another ant-mimicking spider, *Synemosyna* sp., (subfamily Synagylinae) also has significantly reduced levels of cuticular hydrocarbons relative to its model ant (D. Uma, unpublished data). Having low amounts of hydrocarbons (chemical insignificance) is a strategy commonly used by nest parasites to escape detection when entering host colonies (ants [40]–[41], social wasps [42]). Although our results are consistent with an interpretation of chemical insignificance, further studies are needed to test this and other possible chemical mechanisms, including lack of specific cuticular compounds, and to assess the relative contributions of chemistry and behavior to the reduced aggression and predation experienced by *Peckhamia*.

### Multimodal cues for multiple audiences

Animal displays often involve complex signaling across multiple sensory modalities [43]. Use of multimodal signaling is often seen in aposematic or warning displays [44], in courtship displays [45]–[46] or in deception [1]. Most of these studies, however, focus on a specific target audience, such as a potential predator or a mate, which responds to such multimodal signals.

To our knowledge, a mimic's use of two signal modalities, each deceiving a different audience, has not been previously reported. We have previously shown that *Peckhamia* gains protection from visually-oriented enemies, and in the current study, our behavioral results provide clear evidence that *Peckhamia* also eludes chemically-oriented predators, regardless of the mechanism(s) by which this protection takes place. We believe that the utilization of multimodal cues to deceive multiple audiences is likely to be common, but has yet to be explored in mimicry systems, in part because we visually-oriented humans have not looked beyond the often spectacularly accurate displays of visual mimicry. Understanding multimodal signaling will reveal different selective pressures under which such cues have evolved, and will help us understand the evolution of the form and function of complex signals.

## Supporting Information

File S1 Contains the files: Text S1: Calculation of surface area of ants and spiders. Table S2: Response of focal ants toward test subjects: Nestmate ants and ant mimicking spiders were bitten less compared to non-nestmate ants and non-mimic spiders. Not all contacts resulted in bites. Table S3: Chemical composition of hexane extracts of *Camponotus nearcticus*, containing 43 compounds, with carbon chain length between 25– 31. LRI, linear retention index; sym = symmetric molecule, reduced number of diagnostic ions. Table S4: Chemical composition of subadult *Peckhamia picata*, containing 74 compounds, with carbon chain length between 22–33. LRI, linear retention index; sym = symmetric molecule, reduced number of diagnostic ions.
(DOCX)
