# Supplementary material for: Double Deception: Ant-Mimicking Spiders Elude Both Visually- and Chemically-Oriented Predators
Source: PLoS One. 2013 Nov 13;8(11):e79660. doi: 10.1371/journal.pone.0079660 (PMC3827452; doi:10.1371/journal.pone.0079660)
Supplement: File S1 — Contains the files: Text S1: Calculation of surface area of ants and spiders. Table S2: Response of focal ants toward test subjects: Nestmate ants and ant mimicking spiders were bitten less compared to non-nestmate ants and non-mimic spiders. Not all contacts resulted in bites. Table S3: Chemical composition of hexane extracts of Camponotus nearcticus, containing 43 compounds, with carbon chain length between 25– 31. LRI, linear retention index; sym = symmetric molecule, reduced number of diagnostic ions. Table S4: Chemical composition of subadult Peckhamia picata, containing 74 compounds, with carbon chain length between 22–33. LRI, linear retention index; sym = symmetric molecule, reduced number of diagnostic ions. (DOCX) [file pone.0079660.s001.docx]

Text S1: Calculation of surface area of ants and spiders

For a calculation of the cuticular surface of spiders and ants, we measured length, width and height of head, thorax or (cephalothorax) and abdomen to the nearest mm using a digital caliper. The surface was calculated according to Kroiss et al. [36]. Since abdomen and thorax resemble prolate ellipsoids, the surface was calculated according to A= 2πa^2^b/√a^2^-b^2^[b/a^2^*√a^2^-b^2^+arcsin{√a^2^-b^2^}], where a is semi-major axis (length/2) and b is the semi-minor axis (width+height/4). Cephalothorax or head surface was calculated according to the surface of a cylinder (2π(r+h)), where r is (width+length/4) and h is the length of the head. Finally the surface area of each body part was summed.

Table S2: Response of focal ants toward test subjects: Nestmate ants and ant mimicking spiders were bitten less compared to non-nestmate ants and non-mimic spiders. Not all contacts resulted in bites.

|  | Mean number of bites per contact (SE) | No of trials in which bites occurred following contact | Range of bites per contact |
| --- | --- | --- | --- |
| Nestmate ant | 0.22 (0.13) | 3 | 0-2 |
| Non-nestmate ant | 2.83 (0.55) | 15 | 0-9 |
| Mimic spider | 0.025 (0.024) | 1 | 0-0.4 |
| Non-mimic spider | 2.32 (0.71) | 11 | 0-10 |

Table S3: Chemical composition of hexane extracts of *Camponotus nearcticus*, containing 43 compounds, with carbon chain length between 25- 31. LRI, linear retention index; sym=symmetric molecule, reduced number of diagnostic ions.

| **Substance Name** | **Diagnostic Ions** | **LRI** |
| --- | --- | --- |
| Pentacosane | 352 | 2500 |
| 2-Methylpentacosane | 323, 351 (M-15), 366 (M+) | 2563 |
| Hexacosane | 366 | 2600 |
| 14-Methylhexacosane | 196/197, 210/211, 365 (M-15), 380 (M+) | 2632 |
| 2-Methylhexacosane | 336/337, 365 (M-15), 380 (M+) | 2664 |
| 2,16-Dimethylhexacosane  + 2,14-Dimethylhexacosane | 168/169, 252/253, 379  196/197, 224/225, 379 | 2694 |
| Heptacosane | 380 | 2700 |
| Unknown 1 |  | 2722 |
| 13-Methylheptacosane + 11-Methylheptacosane | 196/197, 224/225, 379 (M-15), 394 (M+);  168/169, 252/253, 379 (M-15), 394 (M+) | 2731 |
| 7-Methylheptacosane | 112/113, 308/309, 379 (M-15) | 2740 |
| 5-Methylheptacosane | 84/85, 336/337 | 2749 |
| 11,15-Dimethylheptacosane | 168/169, 196/197, 238/239, 266/267, 408 (M+) | 2760 |
| 3-Methylheptacosane | 365 (M-29), 379 (M-15) | 2773 |
| 5,15-Dimethylheptacosane | 84/85, 196/197, 238/239, 351 | 2779 |
| Unknown 2 |  | 2793 |
| Octacosane | 394 | 2800 |
| 3,15-Dimethylheptacosane | 196/197, 238/239, 379 | 2814 |
| 3,7,11-Trimethylheptacosane | 126/127, 196/197, 252/253, 322/323, 393 | 2831 |
| Unknown 3 |  | 2845 |
| Unknown 4 |  | 2857 |
| 2-Methyloctacosane | 365, 393 (M-15), 408 (M+) | 2863 |
| 4,12-Dimethyloctacosane | 70/71, 196/197, 252/253, 378/379, 407 (M-15) | 2888 |
| 2,16-Dimethyloctacosane  + 2,14-Dimethyloctacosane | 168/169, 252/253, 407  196/197, 224/225, 407 | 2893 |
| Nonacosane | 408 | 2900 |
| 4,8,12-Trimethyloctacosane | 71, 140/141, 210/211, 252/253, 323, 393, 421 | 2914 |
| Unknown 5 |  | 2919 |
| 15-Methylnonacosane + 13-Methylnonacosane + 11-Methylnonacosane | 224/225 sym, 407 (M-15), 422 (M+); 196/197, 252/253, 407 (M-15), 422 (M+); 168/169, 280/281, 407 (M-15), 422 (M+) | 2930 |
| 7-Methylnonacosane | 112/113, 336/337, 407 (M-15) | 2942 |
| 13,17-Dimethylnonacosane | 196/197, 266/267 sym | 2958 |
| 5,12-Dimethylnonacosane | 84/85, 196/197, 266/267, 379, 421 (M-15) | 2978 |
| Unknown 6 |  | 2987 |
| Unknown 7 |  | 3003 |
| Unknown 8 |  | 3033 |
| Unknown 9 |  | 3054 |
| Unknown 10 |  | 3071 |
| Hentriacontane | 436 | 3100 |
| 13,17-Dimethylhentriacontane | 196/197, 224/225, 266/267, 294/295, 449 (M-15) | 3152 |
| Unknown 11 |  | 3166 |

Table S4: Chemical composition of subadult *Peckhamia picata*, containing 74 compounds, with carbon chain length between 22-33. LRI, linear retention index; sym=symmetric molecule, reduced number of diagnostic ions.

| **Substance Name** | **Diagnostic Ions** | **LRI** |
| --- | --- | --- |
| Docosane | 310 | 2200 |
| 2-Methyldocosane | 280/281, 309 (M-15) | 2262 |
| Tricosene | 322 | 2273 |
| Tricosene | 322 | 2280 |
| Tricosene | 322 | 2290 |
| Tricosane | 324 | 2300 |
| 3-Methyltricosane | 57, 308/309, 323 (M-15) | 2373 |
| Tetracosane | 338 | 2400 |
| 2-Methyltetracosane | 308/309, 336/337 (M-15), 352 (M+) | 2466 |
| Pentacosene | 350 | 2477 |
| Pentacosene | 350 | 2484 |
| Pentacosene | 350 | 2494 |
| Pentacosane | 352 | 2500 |
| 5-Methylpentacosane | 84/85, 308/309, 351 (M-15) | 2550 |
| 2-Methylpentacosane | 323, 351 (M-15), 366 (M+) | 2563 |
| 3-Methylpentacosane | 337, 351 (M-15) | 2573 |
| Hexacosane | 366 | 2600 |
| 2-Methylhexacosane | 336/337, 365 (M-15), 380 (M+) | 2664 |
| 3-Methylhexacosane | 350/351, 365 (M-15) | 2673 |
| Heptacosene | 378 | 2676 |
| Heptacosene | 378 | 2683 |
| 2,16-Dimethylhexacosane  + 2,14-Dimethylhexacosane  + Heptacosene | 168/169, 252/253, 379  196/197, 224/225, 379  378 | 2694 |
| Heptacosane | 380 | 2700 |
| Unknown 12 |  | 2709 |
| 13-Methylheptacosane + 11-Methylheptacosane + 9-Methylheptacosane | 196/197, 224/225, 379 (M-15);  168/169, 252/253, 379 (M-15);  140/141, 280/281, 379 (M-15) | 2731 |
| 7-Methylheptacosane | 112/113, 308/309, 379 (M-15) | 2740 |
| 5-Methylheptacosane | 84/85, 336/337, 379 (M-15) | 2749 |
| 2-Methylheptacosane | 351, 379 (M-15) | 2763 |
| 3-Methylheptacosane | 365 (M-29), 379 (M-15) | 2773 |
| Octacosane | 394 | 2800 |
| 14-Methyloctacosane + 13-Methyloctacosane + 12-Methyloctacosane + 11-Methyloctacosane + 9-Methyloctacosane | 210/211, 224/225, 393 (M-15);  196/197, 238/239, 393 (M-15);  182/183, 252/253, 393 (M-15);  168/169, 266/267, 393 (M-15);  140/141, 295, 393 (M-15) | 2830 |
| 2-Methyloctacosane + Nonacosadiene | 365, 393 (M-15), 408 (M+);  404 | 2863 |
| Nonacosene | 406 | 2884 |
| 2,16-Dimethyloctacosane  + 2,14-Dimethyloctacosane | 168/169, 252/253, 407  196/197, 224/225, 407 | 2893 |
| Nocacosane | 408 | 2900 |
| 15-Methylnonacosane + 13-Methylnonacosane + 11-Methylnonacosane | 224/225 sym, 407 (M-15);  196/197, 252/253, 407 (M-15);  168/169, 280/281, 407 (M-15) | 2930 |
| 9-Methylnonacosane | 140/141, 308/309, 407 (M-15) | 2936 |
| 7-Methylnonacosane | 112/113, 336/337, 407 (M-15) | 2942 |
| 5-Methylnonacosane | 84/85, 364/365, 407 (M-15) | 2949 |
| 11,15-Dimethylnonacosane + 13,17-Dimethylnonacosane | 168/169, 224/225, 238/239, 295, 421 (M-15);  196/197, 266/267 sym | 2956 |
| 2-Methylnonacosane | 71, 379, 407 (M-15) | 2962 |
| 3-Methylnonacosane | 56/57, 393 (M-29), 407 (M-15) | 2974 |
| 5,15-Dimethylnonacosane + 5,12-Dimethylnonacosane | 84/85, 224/225, 238/239, 379, 421 (M-15); 84/85, 196/197, 266/267, 379, 421 (M-15) | 2978 |
| Triacontane | 422 | 3000 |
| 10-Methyltriacontane | 154/155, 308/309, 421(M-15) | 3032 |
| 2-Methyltriacontane | 393, 421 (M-15) | 3063 |
| Hentriacontane | 436 | 3100 |
| 15-Methylhentriacontane + 13-Methylhentriacontane + 11-Methylhentriacontane + 9-Methylhentriacontane | 224/225, 252/253, 435 (M-15);  196/197, 280/281, 435 (M-15);  168/169, 308/309, 435 (M-15);  140/141, 336/337, 435 (M-15) | 3130 |
| 11,15- Dimethylhentriacontane + 13,17-Dimethylhentriacontane | 168/169, 238/239, 252/253, 322/323, 449 (M-15);  196/197, 224/225, 266/267, 294/295, 449 (M-15) | 3153 |
| 3-Methylhentriacontane | 56/57, 421 (M-29), 435 (M-15), 450 (M+) | 3175 |
| Dotriacontane | 450 | 3200 |
| 17-Methyltritriacontane + 15-Methyltritriacontane + 13-Methyltritriacontane + 11-Methyltritriacontane + 9-Methyltritriacontane | 252/253 sym;  224/225, 280/281;  196/197, 308/309;  168/169, 336/337;  140/141, 365 | 3318 |
